# Native myocardial T1 is elevated in subjects with coronary microvascular dysfunction and no obstructive CAD

**DOI:** 10.1186/1532-429X-17-S1-P141

**Published:** 2015-02-03

**Authors:** Jaime L Shaw, Janet Wei, Puja K Mehta, David Chen, Michael Nelson, Louise Thomson, Daniel S Berman, Noel C Bairey Merz, Debiao Li, Behzad Sharif

**Affiliations:** Biomedical Imaging Research Institute, Cedars-Sinai Medical Center, Los Angeles, CA USA; Bioengineering, University of California, Los Angeles, Los Angeles, CA USA; Cedars-Sinai Heart Institue, Los Angeles, CA USA; Biomedical Engineering, Northwestern University, Chicago, IL USA

## Background

Women with signs and symptoms of ischemia but no obstructive coronary artery disease (CAD), often have coronary microvascular dysfunction (CMD) evidenced by the Women's Ischemia Syndrome Evaluation (WISE) studies [1, 2]. CMD is associated with higher risk of adverse cardiac events including heart failure compared to healthy women [1, 2]. Elevated native T1 values are known to be suggestive of myocardial fibrosis. We hypothesized that the native myocardial T1 would be abnormally elevated indicating fibrosis in WISE subjects with CMD.

## Methods

CMD subjects (n=14) from symptomatic women with objective evidence of myocardial ischemia enrolled in the NHLBI-sponsored WISE studies were evaluated. Subjects with evidence of obstructive CAD (defined as ≥ 50% epicardial stenosis in at least one artery), left ventricular dysfunction, left ventricular hypertrophy, or valvular/structural heart disease were excluded.

T1 mapping using a vendor-provided MOLLI sequence for the mid-ventricular slice was performed at 1.5T (Magnetom Avanto, Siemens Healthcare). The MOLLI acquisitions were ECG-triggered and obtained during an 11-heartbeat breath-hold and T1 maps were generated following on-line automatic motion correction [3, 4]. The mean myocardial T1 value for each subject was measured by a standard myocardial segmentation scheme. For comparison, the average native T1 value for normal subjects (n=62) as previously reported in the literature [5] was used, all measured using the same vendor-provided MOLLI sequence on the same type of scanner (1.5T Magnetom Avanto). The native T1 values for women with CMD were compared to reported normal controls using a one-sample t-test.

## Results

**Figure 1:**
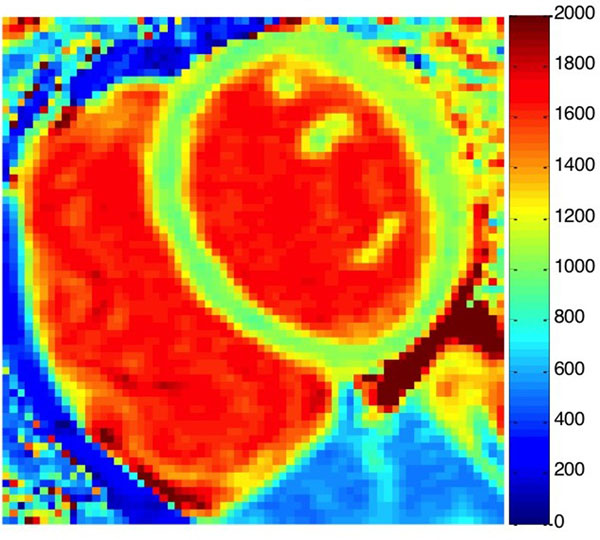
Native T1 map from a WISE subject with CMD with average native T1 of 1060.6±69.3 ms.

**Figure 2:**
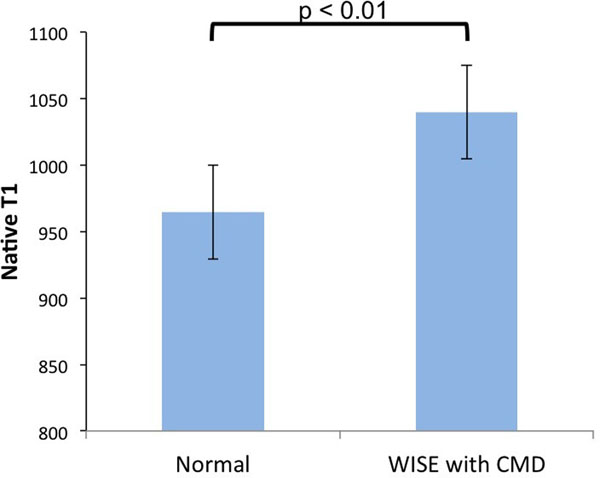
Native myocardial T1 for WISE subjects with CMD are higher compared with normal controls (mean values: 1039.8 ms versus 964.6 ms, p < 0.01).

The mean age of WISE subjects with CMD was 57±11 years with an average BMI of 23.9; 34.7% had hypertension, 7.1% had diabetes. All subjects had preserved ejection fraction (mean EF: 63%). No focally elevated native T1 region was observed in any of the subjects. A representative T1 map is shown in Figure 1. The reported normal group was 51.6% female with an average age of 43.6±17.4 and average BMI of 26.5. The mean native myocardial T1 values in women with CMD were higher compared with normal subjects (1039.8 ms ± 35.1 versus 964.6 ± 35.3, p < 0.01), shown in Figure 2.

## Conclusions

Native T1 values in women with signs and symptoms of ischemia and no obstructive CAD were significantly elevated compared with normal values reported in the literature. Our initial findings suggest presence of diffuse myocardial fibrosis in this population. Future studies using age and gender matched normal controls are needed to confirm these initial findings. The presence of diffuse myocardial fibrosis may elucidate a potential underlying mechanism leading to heart failure and other adverse events in patients with CMD, with important therapeutic implications.

## Funding

Grant sponsors: NIH National Heart, Lung and Blood Institute grant nos. K99 HL124323-01 and R01 HL090057-01; and the Barbra Streisand Women's Cardiovascular Research & Education Program, CSMC.
